# Social Determinants of Health in Maternity Care: A Quality Improvement Project for Food Insecurity Screening and Health Care Provider Referral

**DOI:** 10.1089/heq.2020.0120

**Published:** 2021-09-14

**Authors:** Chelsea D. Fitzhugh, Marina Stranieri Pearsall, Kristin P. Tully, Alison M. Stuebe

**Affiliations:** University of North Carolina School of Medicine, Chapel Hill, North Carolina, USA.

**Keywords:** social determinants of health, food insecurity, maternity care, screening, referral

## Abstract

**Purpose:** This quality improvement project evaluated implementation of social determinants of health screening and referral for food insecurity.

**Methods:** Four obstetric providers used study-developed resources to screen and refer English-speaking patients (*n*=14) during clinic visits. Providers and patients completed post-visit questionnaires. Provider feedback informed improvements to the intervention approach for consecutive study shifts.

**Results:** Providers and patients reported high satisfaction with encounters. Referrals were made for four patients. Challenges to implementation included resource organization, time constraints, and integration into clinic workflow.

**Conclusion:** Processes for universal screening and tailored information provision are areas to continue to strengthen for establishing equitable health care.

## Introduction

Patient-centered health care increasingly includes screening and referral for social determinants of health (SDoH), which are structural and social drivers of health outcomes.^1^ Obstetric health care teams are recommended to screen and refer to support two generations of patients.^2^ Although there is opportunity for promoting health equity through addressing SDoH, incorporating screening and referral into clinical workflow is challenging.

Existing quality improvement (QI) work on SDoH screening and referral has primarily been conducted in other fields of medicine. In pediatric care, a QI team consisting of providers, staff, and parents established practice-level protocols for SDoH screening and referral management.^3^ QI teams routinely sought feedback by collecting surveys from clinic team members and parent/caregivers and by meeting with community partners to identify available resources.^3^ Challenges to the follow-up of positive screens included limited availability of internal resources and communication barriers between practices and community agencies.

Less is known about how to best screen and refer for SDoH specifically in maternity care, which may be complicated by patient perceptions of stigma associated with pregnancy-related resource utilization^4^ and patient perceptions of providers' bias^5,6^ when race- or class-based assumptions may be made.^7^ Although North Carolina maternity care providers have SDoH screening forms available, with care coordination guidance and reimbursement such as through the Community Care of North Carolina,^8^ implementation support does not include how or when clinicians might best communicate with patients and make referrals. SDoH engagements are opportunities to share the full scope of health care services with patients, normalize challenges to health care utilization, and reassure patients, thus offering resources while earning trust and healing. To build on this knowledge in maternity care, we sought to test strategies for SDoH screening and referral in an obstetric care clinic.

## Methods

This study was reviewed by the University of North Carolina Biomedical IRB (No. 18-2811) and determined to be exempt. This QI project was the final phase of a mixed-methods research project on SDoH within maternity care. For the full study, we used clinical shadowing, interviews, focus groups, and in-person workshops with patients and clinicians to determine and prioritize components of sustainable SDoH screening and referral. Building on this formative work, we conducted this QI project within a large U.S. teaching hospital clinic from September to December 2019. We focused this aspect of the research on workflow around addressing food insecurity because on-site food packages and community resource existed to address this need.

**FIG. 1.**
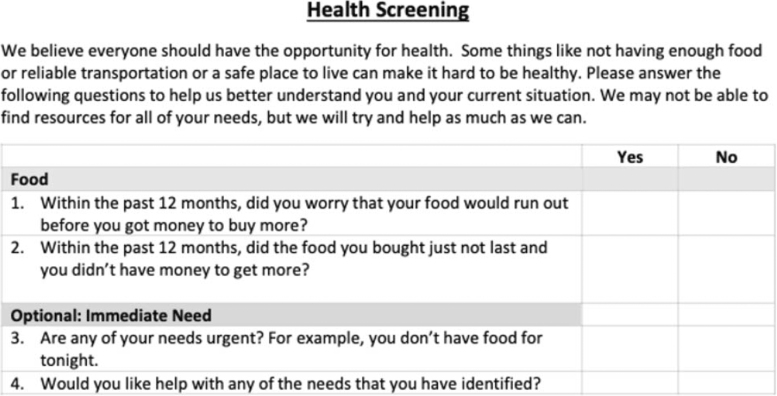
Food insecurity screening. Patient participants were asked to complete the food insecurity screening before their clinic visit.

Patients were eligible if they spoke English, were at least 18 years of age, and were being seen by a participating health care provider. Participating providers and patients were verbally consented. This project was completed over the course of four clinical shifts, each involving three to five patients. Each shift involved a separate provider integrating screening and referral into routine care and study questionnaire completion. Before their clinic visit, each patient participant completed a food insecurity screening with the purpose of the questions framed (Fig. 1). Screening responses were only offered to the health care providers, not collected as study data. The focus of the study was to advance SDoH screening and referral into clinical workflows, not assess the extent of unmet patient needs.

Before each clinic shift, provider participants received a document offering tips for engaging around SDoH with patients (Supplementary Appendix SPA1) and a document listing food resources in four counties served by the clinic (Supplementary Appendix SPA2). Providers were asked to review patient food insecurity screening and to refer patients as clinically indicated.

Patients completed questionnaires at the end of their visit. Providers completed similar questionnaires at the end of each study-associated clinic shift (Supplementary Appendix SPA3). These questions were structured to evaluate perspectives on how well providers addressed patients' needs, perspectives on patient–provider communication, and satisfaction. Questions included the validated patient satisfaction survey,^9^ where responses were recorded on a visual analog scale ranging from 0 (not at all) to 100 (extremely). After each session, the data collected from patient and provider participants were used to identify screening and referral barriers and improve the process. Participant characteristics were summarized using descriptive statistics and the visual analog scores are presented in bar charts.

## Results

### Sample characteristics

Characteristics of the patient sample (*n*=14) are shown in Table 1. The four participating clinicians were non-Hispanic white and included two physicians, a nurse practitioner, and a certified nurse-midwife.

**Table 1.** Patient Sample Characteristics (*N*=14)

	*n* (%)
Ethnicity and race	
Hispanic	2 (14.3)
Non-Hispanic	12 (85.7)
Black/African American	5 (35.7)
White	4 (28.6)
Black/African American and white	3 (21.4)
Other (unspecified)	1 (7.1)
Unknown	1 (7.1)
Insurance types	
Medicaid	12 (85.7)
Private	6 (42.9)
Other^*^	1 (7.1)
Appointment type	
Follow-up prenatal visit	10 (71.4)
First prenatal visit	2 (14.3)
Postpartum or gynecological care	2 (14.3)

“Insurance Types” percentages sum to greater than 100% due to some responders indicating more than one category.

^*^ Other includes Tricare, Blue Cross Blue Shield and Medicare.

### Analysis of screening and referral implementation

Health care provider feedback identified several workflow efficiency challenges. In the first shift, the provider reported “information overload.” This provider was given an SDoH tip sheet, community food resource document, and a provider questionnaire for each of their patient visits. To streamline the printed materials, the providers in future shifts were given one SDoH tip sheet, one research questionnaire, and multiple copies of the food resource documents for potential distribution to patients.

Additional provider feedback questioned the study provision of parking vouchers as the only form of patient compensation. Some patients do not drive to their appointments, so the study incentives were modified to include the option for hospital meal vouchers.

Some logistical challenges uncovered problems beyond the scope of solutions that could be addressed through the QI project. For example, provider feedback from the third shift revealed difficulty with integrating SDoH care within time constraints. A full clinic schedule with patients with complex health needs called attention to the importance of future strengthening of care coordination.

**FIG. 2.**
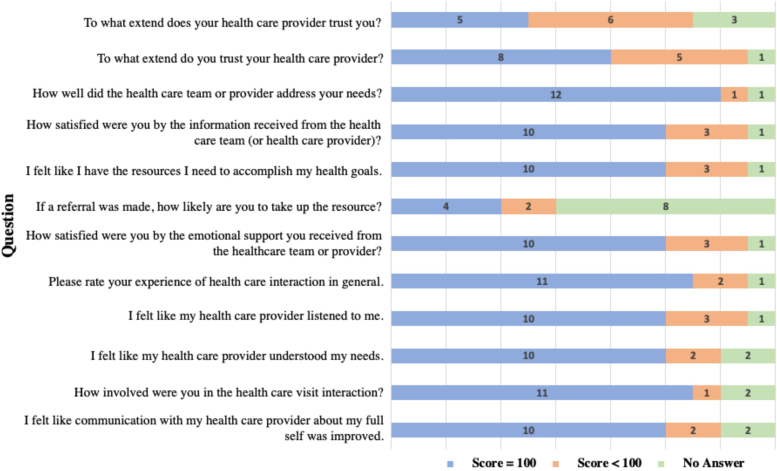
Patient questionnaire (*N*=14). Response scores were entered on a visual analog scale ranging from 0=“not at all” to 100=“extremely.”

**FIG. 3.**
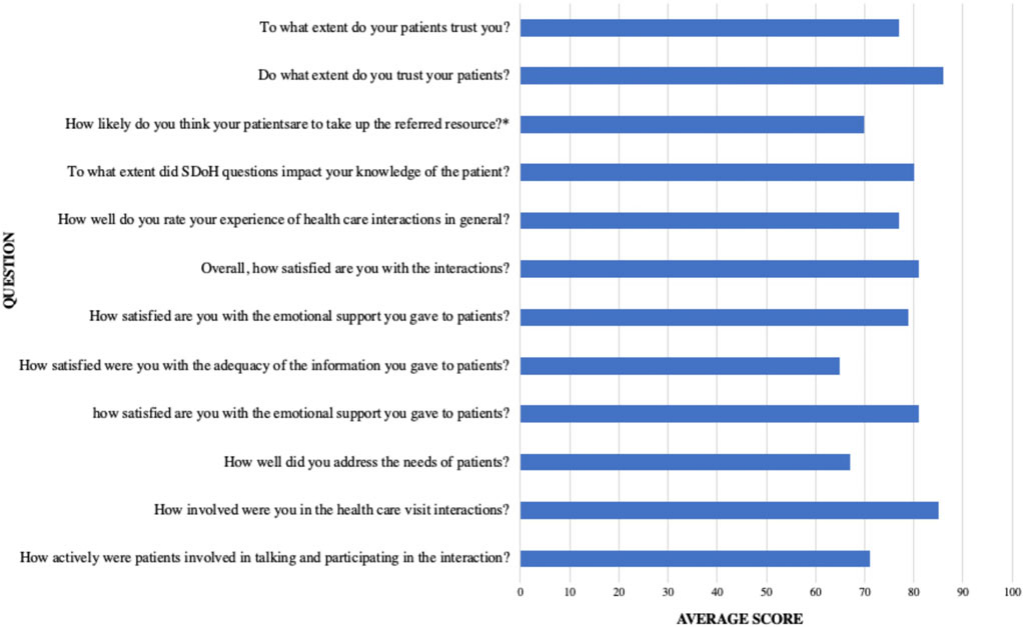
Health care provider questionnaire (*N*=4). Response scores were entered on a visual analog scale ranging from 0=“not at all” to 100=“extremely.” *Average of three recorded responses.

Most patients were satisfied with their visits and responded positively regarding the patient–provider relationship (Fig. 2). Providers also perceived screening and referral positively (Fig. 3). In open-ended responses, two providers noted that they did not expect positive screens for food insecurity in their patient cohort. Providers reported that four patients screened positive for food insecurity and three of these individuals were provided with resource referrals during the visits. One of these patients identified urgent food needs and was provided with a food package on-site. The fourth positive screen was not identified until after the patient left the facility because the provider did not review the screening responses until that point. To mitigate future risk of not reading screens before or during encounters, the screening form was printed on brightly colored paper to signpost its importance. Additional provider recommendations to improve care included social worker presence during encounters, provider training on referral options, and the clinic offering patients funds for food.

## Discussion

This QI project suggests value of routine, universal SDoH screening and referral while highlighting the implementation challenges in an OBGYN clinic. Challenges included resource organization, time constraints, and integration into workflow, although most patients and providers were satisfied with their clinical encounters. The majority of patients reported feeling heard and emotionally supported by their providers, which is foundational for quality, respectful health care.^10^

SDoH profoundly affects patients' health outcomes as well as their experiences with and within the health care system. Food insecurity in particular impacts the health of pregnant and parenting patients. It has been associated with elevated levels of depressive symptoms.^11^ Maternity care is a valuable opportunity to establish positive health care engagement for two patients, the parent and the infant. High-quality care can integrate patients into the health care system during pregnancy and beyond. Provider communication skills and empathy are important for patient outcomes and perspectives of their health care team and the system.^12–15^

Similar to other research findings, we identified time as a significant barrier to clinically addressing SDoH.^16^ There is a need for ongoing SDoH support, in which needs are addressed over a series of visits.^17^ Encounters might be restructured to accommodate more time for care coordination with social work and health care navigators. In addition, changes to reimbursement options to include coverage to otherwise unbillable services will enable health care teams to increase precision medicine and care value.

Strengths of our study include analysis of questionnaire data alongside process feedback with incremental modifications of our intervention. Furthermore, we limited scope to a single SDoH to avoid uncovering problems without the ability to address them. Equitable care requires honest patient sharing and health care facility readiness to provide timely and clear information for any and all resources a patient might need.

Our findings should be interpreted within the context of the study design. This was a small QI project within a single site. Future studies might explore SDoH screening and referral processes in clinics with varying patient demographics and support staff presence or focus on varying patient perspectives among prenatal versus postpartum visits. The challenge of “information overload” during a single clinic visit is important to consider from both clinician and patient perspectives. In addition, providing customized materials to patients is difficult with printed documents, so leveraging emerging online resources for state-wide and local SDoH supports may be helpful to efficiently provide actionable information to patients.^18^

## Conclusion

Results from this study add to existing literature supporting the potential positive impacts of incorporating SDoH screening and referral into maternity care. Our project offers insights into framing SDoH screening and practical steps to add value to the clinical encounter. When implementing new tools, determining and then centering action around the needs of end users is vital for effective and sustained impact.

## Supplementary Material

Supplemental data

Supplemental data

Supplemental data

## Supplementary Material

Supplementary Appendix SA1

Supplementary Appendix SA2

Supplementary Appendix SA3

## Abbreviations Used

QI: quality improvement
SDoH: social determinants of health
