# Supplementary material for: Social Determinants of Health in Maternity Care: A Quality Improvement Project for Food Insecurity Screening and Health Care Provider Referral
Source: Health Equity. 2021 Sep 14;5(1):606–11. doi: 10.1089/heq.2020.0120 (PMC8665789; doi:10.1089/heq.2020.0120)
Supplement: Supplemental data [file Suppl_AppendixSA1.docx]

**Appendix 1. SDoH Provider Tip Sheet**
