# Supplementary material for: Social Determinants of Health in Maternity Care: A Quality Improvement Project for Food Insecurity Screening and Health Care Provider Referral
Source: Health Equity. 2021 Sep 14;5(1):606–11. doi: 10.1089/heq.2020.0120 (PMC8665789; doi:10.1089/heq.2020.0120)
Supplement: Supplemental data [file Suppl_AppendixSA2.pdf]

County/ies Served	Organization Name/Address/ Phone Number	Operational Hours	Intake/Qualifications/Food Services Provided	Other Support Offered	Nearest public transportation
**Alamance, Durham, Chatham, Orange**	**Binkley Baptist Church**, 1712 Willow Dr, Chapel Hill<br>919-942-4964	Mon-Fri, 9am-3pm	-Delivers prepared meals to homebound individuals<br><br>-Free<br><br>-Appointment required	-Rent and mortgage payment assistance	Bus: University Place<br>Transit: Willow Dr at Willow Terrace Condos
