# Supplementary figures and images for: Social Determinants of Health in Maternity Care: A Quality Improvement Project for Food Insecurity Screening and Health Care Provider Referral
Source: Health Equity. 2021 Sep 14;5(1):606–11. doi: 10.1089/heq.2020.0120 (PMC8665789; doi:10.1089/heq.2020.0120)

**Provider Questionnaire**


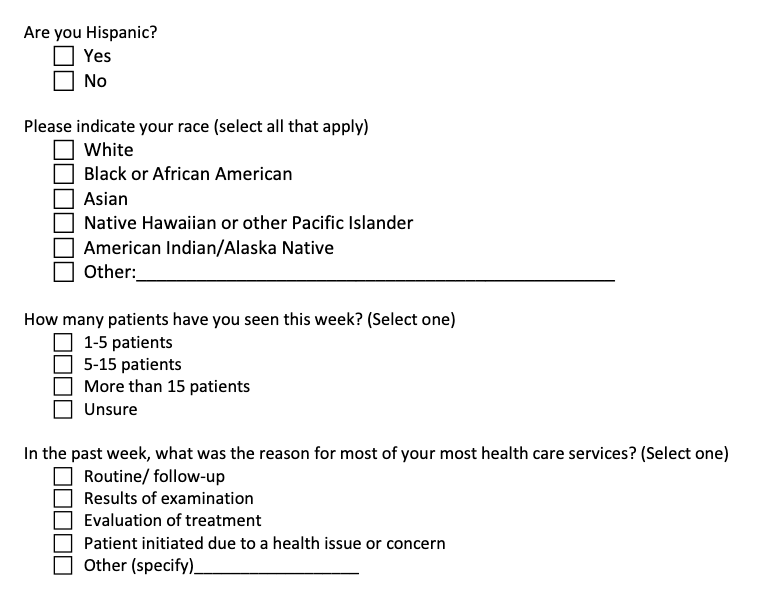


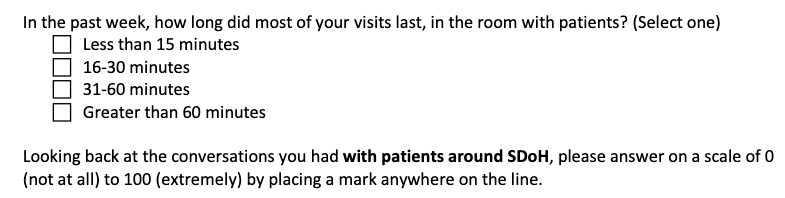


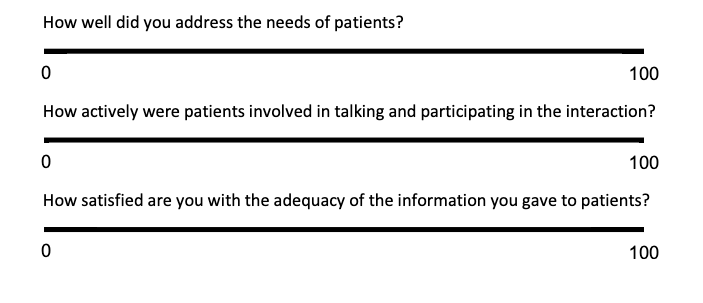


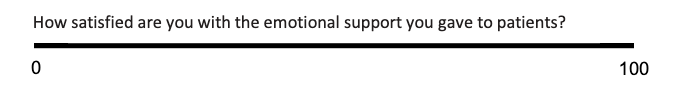


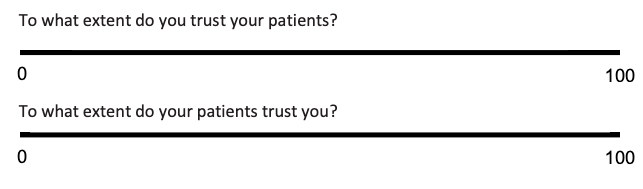


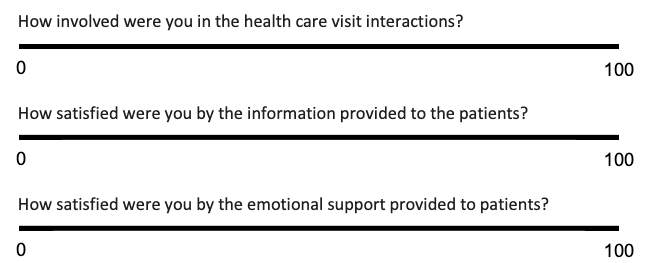

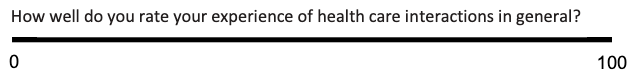


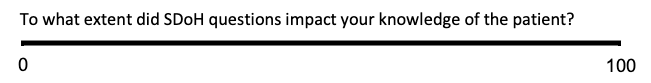


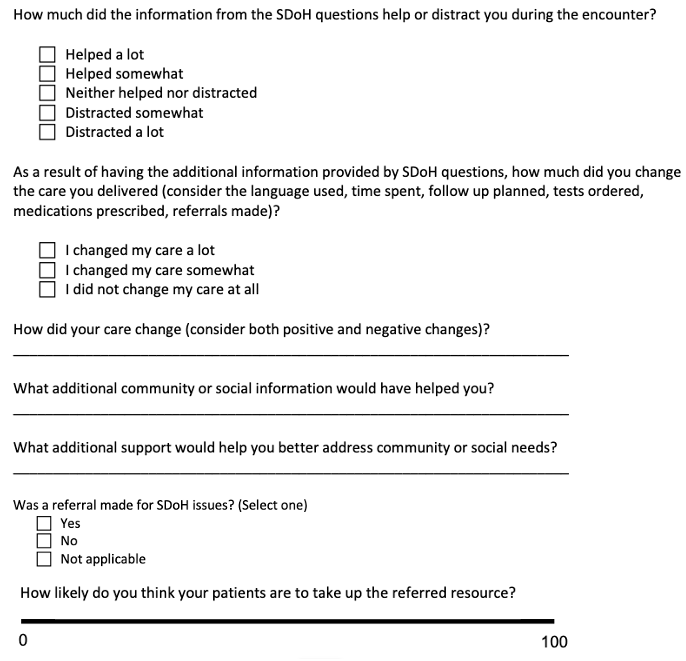

Supplement: Supplemental data [file Suppl_AppendixSA3.docx]
